# Inoculation, co-inoculation, and re-inoculation with *Rhizobium tropici* and *Azospirillum brasilense*: effects on nodulation, growth, and agronomic performance of common bean

**DOI:** 10.3389/fpls.2026.1815474

**Published:** 2026-07-17

**Authors:** Nathan Mickael de Bessa Cunha, Itamar Rosa Teixeira, Gisele Carneiro da Silva Teixeira, Ednaldo Cândido Rocha, Antonio Alannilson Neres de Oliveira, Andressa Laís Caldeira de Souza, Tamires Ester Peixoto Bravo, Alexandre Marcos Sbroggio Filho

**Affiliations:** 1 Institute of Agricultural Sciences, Goiás State University, Anápolis, GO, Brazil; 2 Goiás State University, Ipameri, GO, Brazil; 3 Department of Agricultural Engineering, Federal University of Viçosa, Viçosa, MG, Brazil; 4 Department of Agronomy, Federal University of Goiás, Goiânia, GO, Brazil

**Keywords:** bioinputs, BNF, nutrition, *Phaseolus vulgaris*, soil fertility, yield

## Abstract

The use of inoculants to meet nitrogen demand in common bean crops is still limited among farmers. However, strategies such as co-inoculation and top-dressing re-inoculation have emerged as promising alternatives capable of improving nodulation and reducing mineral nitrogen dependency in common bean. This study aimed to evaluate the effects of different inoculation techniques, co-inoculation with *Rhizobium tropici* and *Azospirillum brasilense*, and top-dressing re-inoculation on the morphophysiological and agronomic performance of common bean. The experiment was conducted during the 2024/2025 wet season using a randomized block design with four replications and twelve treatments: T1 – Seed inoculation with *R. tropici*; T2 – In-furrow inoculation with *R. tropici*; T3 – Top-dressing inoculation with *R. tropici*; T4 – Seed co-inoculation (*R. tropici* + *A. brasilense*); T5 – In-furrow co-inoculation (*R. tropici* + *A. brasilense*); T6 – Top-dressing co-inoculation (*R. tropici* + *A. brasilense*); T7 – T1 + subsequent top-dressing re-inoculation; T8 – T2 + subsequent top-dressing re-inoculation; T9 – T4 + subsequent top-dressing re-inoculation; T10 – T5 + subsequent top-dressing re-inoculation; T11 – Control without any N source; T12 – Mineral N fertilization at sowing and top-dressing (100 kg ha^-1^ N). Results indicated that co-inoculation and re-inoculation techniques provided the best performance in terms of nodulation, morphophysiological aspects and agronomic characteristics of common bean. Treatments involving co-inoculation of *R. tropici* with *A. brasilense*, regardless of the application method, followed by top-dressing re-inoculation, resulted in grain yields of up to 3,042 kg ha^-1^, a value equivalent to that obtained with mineral nitrogen fertilization. The use of inoculants through co-inoculation combined with top-dressing re-inoculation represents an effective and sustainable strategy for integrated nutrient management, reducing the dependency on nitrogen fertilizers, promoting yield gains, cost reduction, and mitigation of environmental impacts.

## Graphical Abstract

**Figure:**
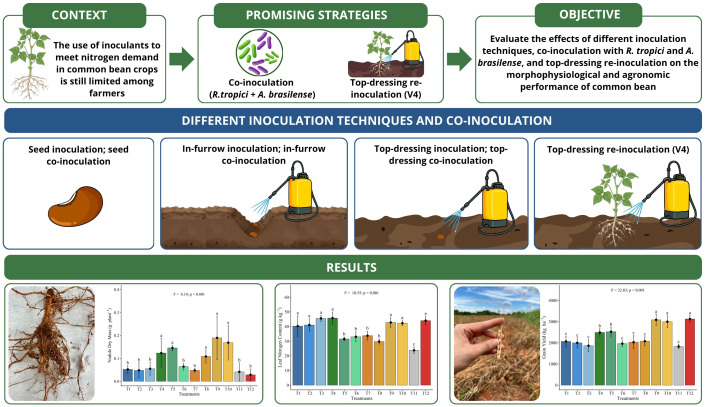

## 1 Introduction

Common bean (*Phaseolus vulgaris* L.) is one of the main grain legumes belonging to the Fabaceae family, widely cultivated in various regions of the world in diverse varieties and colors, especially in Central and Latin America, Africa, and Asia (FAO, 2025). Brazil is the world’s largest producer of the species, with a production of 3,3 million tons of grain (CONAB, 2025). Its grain is of great importance both in strengthening agriculture and in cuisine, being an integral part of the diet, especially in developing countries, due to the significant amounts of proteins, carbohydrates, fibers, vitamins, and minerals present in the grains (Ronko et al., 2021).

Nitrogen (N) is a critical nutrient for the development of most plants, especially in tropical regions, including the common bean, which is considered a poor atmospheric N fixer when compared to other legumes such as soybean (Deresa, 2018). Deficiency causes abiotic stress, resulting in less biomass and alterations in biochemical reactions and soil interactions (Rosolem et al., 2017; Momesso et al., 2019; Basnet et al., 2022).

In general, the nitrogen recommendation for common bean varies from 60 to 150 kg ha^-1^, split into two applications at sowing and top-dressing. This demand can be met through soil nitrogen, nitrogen fertilization, and the Biological Nitrogen Fixation (BNF) process. BNF has the capacity to contribute, on average, with 65% of the nitrogen absorbed by the crop, significantly reducing the reliance on mineral fertilizers, according to recent research results by (Sousa et al., 2022a; Lourenço et al., 2023; Ribeiro et al., 2024). It is a natural phenomenon performed by diazotrophic bacteria that possess a biological mechanism capable of capturing atmospheric nitrogen, which is not assimilable by plants and transforming it into a form that can be assimilated and participate in the nutrition of agricultural crops, such as legumes (Imran et al., 2021).

Co-inoculation is a management practice used to obtain synergistic benefits by combining rhizobia and Plant Growth-Promoting Bacteria (PGPB), such as *Azospirillum brasilense*. While rhizobia act directly on N supply through nodulation, *A. brasilense* stimulating plant growth through physiological changes resulting from the release of phytohormones such as auxins and cytokinins, which favor root growth increase (Zafar et al., 2012). This root expansion maximizes water and nutrient uptake and induce the formation of root hairs, enabling an increase in dry mass production and the number of nodules per plant (Giri et al., 2025).

Co-inoculation of rhizobia and *Azospirillum* has been reported as beneficial for common bean in several studies. According to (Steiner et al., 2019), there was a notable increase in dry mass accumulation in both shoot and roots compared to non-inoculated plants of common bean cv. Pérola. Furthermore, these authors also found a 43% increase in the number of nodules per plant compared to exclusive inoculation with rhizobia. Peres et al. (2016) observed a 149% increase in bean nodulation when using co-inoculation with *R. tropici* and *A. brasilense* compared to single inoculation with *R. tropici*. Co-inoculation with *R. tropici* and *A. brasilense* resulted in significant improvements in nodulation, morphophysiological and agronomic traits. In addition, this treatment showed the highest grain yield stability, representing an increase of 15.4% compared to inoculation with *R. tropici* alone (Messias et al., 2023b).

Another technique recently investigated by research is the strategy of top-dressing re-inoculation, using *R. tropici* or combined co-inoculation with *R. tropici* + *A. brasilense*. Re-inoculation consists of applying inoculant to the soil surface, directed at the plant’s root system, with the purpose of maintaining the rhizobia population in the soil at adequate levels. Environmental factors such as rainfall or irrigation facilitate the movement of these bacteria into the rhizosphere, aiming to recolonize active nodulation sites and maintain the bacterial population in the soil at adequate levels (Teixeira et al., 2022). Although it is a technique that remains understudied, it represents a promising tool to sustain symbiotic efficiency throughout the crop cycle.

In this context, this study aimed to evaluate the effect of inoculation, co-inoculation, and re-inoculation with *R. tropici* + *A. brasilense* on common bean. The objective is to evaluate the potential of these techniques to improve nodulation, morphophysiological growth, and crop yield, positioning them as an effective strategy for integrated nutrient management (INM) to partially replace or reduce the dependence on mineral nitrogen fertilization.

## 2 Materials and methods

### 2.1 Study area characterization

**Figure 1:**
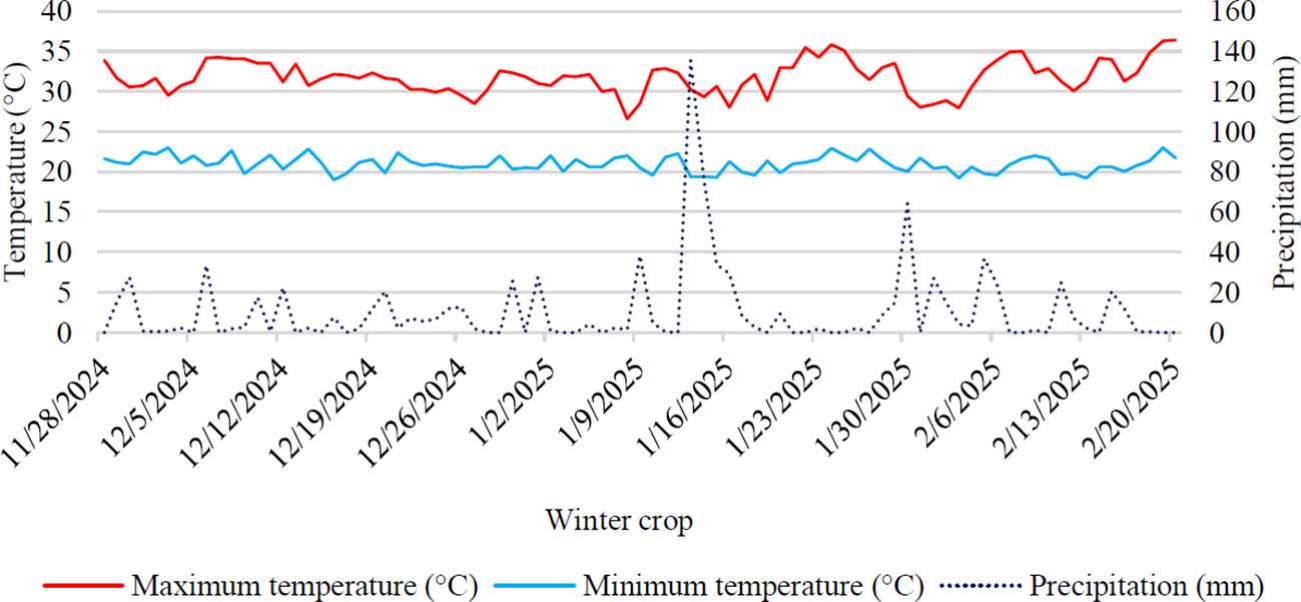
Climatological data for maximum and minimum temperatures and precipitation from November 2024 to February 2025 in the municipality of Anápolis, Goiás State, Brazil (INMET, 2025).

The experiment was conducted during the 2024/2025 wet season at the experimental area of the Goiás Agency for Technical Assistance, Rural Extension, and Agricultural Research (EMATER), located in Anápolis, Goiás, Brazil (16°20’12.13” S, 48°53’15.96” W; altitude 1.058 m) (Google, 2023). The prevailing climatic data during the experiment are presented in **Figure 1**.

Soil samples, classified as Dystrophic Red-Yellow Latosol (*Oxisol*) (Santos et al., 2018), were collected from the 0–0.20 m layer for physicochemical analysis. The results were: pH (CaCl_2_) 4.5; Organic Matter (OM) 31 g dm^-3^; P 7.4 mg dm^-3^; K 92.4 mg dm^-3^; Ca 1.3 cmol_c_ dm^-3^; Mg 0.4 cmol_c_ dm^-3^; Zn 6.7 mg dm^-3^; Cu 3.4 mg dm^-3^; Fe 33.4 mg dm^-3^; Mn 19.6 mg dm^-3^; Na 0.9 mg dm^-3^; Al 0.3 cmol_c_ dm^-3^; H+Al 5.1 cmol_c_ dm^-3^; CEC 7.04 cmol_c_ dm^-3^; and Base Saturation (V%) 27.61%. Granulometric analysis indicated 400, 100, and 500 g kg^-1^ of clay, silt, and sand, respectively.

The experiment was implemented in an area with a long history of corn cultivation for grain in the spring-summer for over ten years, subsequently combined with fallow in the autumn-winter with the forage grass *Brachiaria decumbens*. It is noteworthy that these plants are not nodulating, and therefore microbiological soil analysis was unnecessary, given that the population of bean nodulifying bacteria is low or even non-existent.

### 2.2 Experimental design and treatments

A randomized block design (RBD) was adopted, consisting of 12 treatments and four replications. The treatments involved different inoculation strategies, varying the application methods (seed, planting furrow, and top-dressing) and re-inoculation. The treatments were defined as: T1 – Seed inoculation with *R. tropici*; T2 – In-furrow inoculation with *R. tropici*; T3 – Top-dressing inoculation with *R. tropici*; T4 – Seed co-inoculation (*R. tropici* + *A. brasilense*); T5 – In-furrow co-inoculation (*R. tropici* + *A. brasilense*); T6 – Top-dressing co-inoculation (*R. tropici* + *A. brasilense*); T7 – T1 + subsequent top-dressing re-inoculation; T8 – T2 + subsequent top-dressing re-inoculation; T9 – T4 + subsequent top-dressing re-inoculation; T10 – T5 + subsequent top-dressing re-inoculation; T11 – Absolute control (no N source); T12 – Mineral N fertilization at sowing and top-dressing (100 kg N ha^-1^).

Commercial strains of *R. tropici* for bean (SEMIA 4077 and 4080; 3x10^9^ CFU mL^-1^) were used at the manufacturer’s recommended dose (Nodusoja^®^) of 200 mL per 25 kg of seeds. For *A. brasilense* (Ab-V5 and Ab-V6; 2x10^8^ CFU mL^-1^), the recommended dose was 100 mL per 25 kg of seeds. Doses were doubled, following manufacturer recommendations for new planting areas, to compensate for potential initial abiotic stresses, such as soil acidity, and to ensure the successful colonization and survival of the introduced bacteria against native microbiota (Galindo et al., 2018; Almeida et al., 2022; Rawal et al., 2024). Additionally, four control conditions were tested: control without mineral N, seeds inoculated with *R. tropici* and co-inoculated with *A. brasilense*, and mineral nitrogen fertilization 16 and 84 kg N ha^-1^ at sowing and top-dressing, respectively. No chemical, insecticide, or fungicide treatment was performed on the seeds in order to avoid compromising the viability of the inoculated bacteria.

### 2.3 Crop management

Soil preparation involved the application of dolomitic limestone filler (100% TRNP - Total Relative Neutralizing Power; 1,5 t ha^-1^) to correct acidity and elevate base saturation, creating a more favorable environment for root growth and rhizobial nodulation efficiency, mitigating the negative effects of the initial pH of 4.5. The area was prepared using conventional tillage, including one plowing followed by two harrowings. Basal fertilization was performed in all plots with 400 kg ha^-1^ of 04-30–10 NPK equivalent to 16 kg ha^-^¹ of N, 120 kg ha^-^¹ of P_2_O_5_, and 40 kg ha^-^¹ of K_2_O, fertilizer applied in the planting furrow.

Experimental plots consisted of four 5-meter-long rows spaced 0.5 meters apart, with the two central rows considered the useful area. The common bean cultivar BRS Estilo was sown at a density of 15 seeds per linear meter. Nitrogen management varied according to the treatment: inoculated and/or co-inoculated plots received a starter dose of 16 kg N ha^-1^ at sowing. This low basal N dose is a standard agronomic recommendation to prevent early nitrogen deficiency before the complete establishment and functional activity of the symbiotic process, without inhibiting nodule formation (Teixeira et al., 2022). The mineral nitrogen treatment received 16 kg N ha^-1^ at sowing and 84 kg N ha^-1^ as top-dressing (urea source), split at 25 and 35 days after emergence (DAE).

Phytosanitary control followed Integrated Pest Management (IPM) guidelines. Weed control was performed pre-emergence with fomesafen + fluazifop-p-butyl. For the control of insect pests (*Diabrotica speciosa*, *Bemisia tabaci*, *Spodoptera* spp.) and fungal diseases (anthracnose and leaf spots), foliar applications were carried out using active ingredients such as deltamethrin, azoxystrobin + difenoconazole, chlorothalonil, and profenofos + cypermethrin, according to recommended technical doses. Other cultural practices followed standard recommendations for common bean cultivation.

### 2.4 Preparation of inoculation, co-inoculation, and re-inoculation

Seed inoculation was performed immediately before sowing, ensuring uniform distribution and drying in the shade. For treatments involving in-furrow application and top-dressing, inoculants were diluted in water with a spray volume equivalent to 5 L and applied using a backpack sprayer. Top-dressing re-inoculation was carried out at the V4 phenological stage, strategically timed at the final vegetative stage. This practice precedes the crop’s peak nitrogen demand during flowering and pod filling and which coincides with the decrease in efficiency of bacteria inoculated via seed (Bravo et al., 2025). The spray directed to the plant base and soil surface, subsequent natural precipitation facilitates the movement of bacterial cells into the rhizosphere, aiming to recolonize active nodulation sites and enhance bacterial survival during this critical period.

### 2.5 Evaluated characteristics

#### 2.5.1 Nodulation assessment

Sampling for nodulation assessment occurred at the R6 stage (full flowering). Three plants were collected per experimental unit, excavated using a straight spade in a soil monolith of approximately 0.3 x 0.3 x 0.3 m centered on the plant, to preserve the root system. In the laboratory, roots were carefully washed under running water over a fine-mesh sieve. Active nodules (>1 mm) were detached and counted to determine the number of nodules per plant (NNP) and dried in a forced-air oven at 65 °C for 72 h to obtain the nodule dry mass per plant (NDM).

#### 2.5.2 Morphological characteristics

Evaluations were performed at the full flowering stage (R6). Three representative plants were collected per plot, preserving the integrity of the root system for the following analyses:

Taproot Length (TRL): Determined by measuring the distance from the plant collar to the root tip (root cap).

Plant Height (PH): Measured from the plant collar to the apex of the main stem.

Root Dry Mass (RDM) and Shoot Dry Mass (SDM): Plants were partitioned into shoot and roots. Samples were placed in kraft paper bags and dried in a forced-air circulation oven at approximately 72 ± 1 °C for 72 hours, until constant mass was reached. Subsequently, dry mass was quantified on a precision scale (0.01 g) and expressed in grams per plant.

2.5.3 Physiological Characteristics: Physiological variables were measured *in situ* at the R6 stage, using the third fully expanded trifoliate leaf in good phytosanitary condition, to assess physiological responses under natural cultivation conditions. To minimize diurnal variation and environmental fluctuations, the readings were taken in the morning between 8:00 and 11:00 AM, under good availability of saturating light and ambient temperature and CO_2_ concentration conditions.

Chlorophyll Content (CC): Measured using a portable chlorophyll meter (ClorofiLOG^®^ model CFL 2060), evaluating optical absorbance in chlorophyll a and b ranges. Results were expressed as the Total Chlorophyll Index (Falker Chlorophyll Index - FCI). Analysis was performed in the field by selecting three central leaflets from three distinct plants per plot, using the fully developed trifoliate leaf located at the apex of the main stem.

Gas Exchange: Gas exchange assessments included net photosynthetic rate (A, µmol CO_2_ m^-2^ s^-1^), transpiration rate (E, mmol H_2_O m^-2^ s^-1^), internal CO_2_ concentration (Ci, µmol m^-2^ s^-1^), stomatal conductance (gs, mol m^-2^ s^-1^), and photosynthetically active radiation (PAR, µmol m^-2^ s^-1^). Measurements were taken in the field during the R6 stage on fully expanded leaves located in the upper third of the plants, using an infrared gas analyzer (IRGA, model LCi-SD) under natural irradiance.

Leaf Nitrogen Content (LNC): Twenty fully developed trifoliate leaves were collected from the main stem in the useful area of the plot. Nitrogen content determination followed the Kjeldahl method, as described by (Malavolta et al., 1997).

2.5.3 Agronomic Characteristics: At physiological maturity (R9), desiccation was performed with diquat (Reglone^®^) to ensure uniformity. Ten plants were harvested to determine yield components: number of pods per plant (NPP), number of grains per pod (NGP), 100-grain mass (HGM), and final stand (FS). Grain yield (GY) was obtained by harvesting the two central rows of the useful area, extrapolated to a hectare basis (kg ha^-1^) and corrected to 13% moisture in grains, according to the methodology described by (Almeida et al., 2022).

### 2.6 Statistical analysis

Data were analyzed for normality and homogeneity of variance. Analysis of Variance (ANOVA) was performed, and when significant, means were grouped using the Scott-Knott test (p < 0.05). Multivariate analyses (MANOVA), specifically Canonical Discriminant Analysis (CDA) (Silva, 2016), were performed to integrate plant responses to treatments. All analyses were processed in the R environment (R Core Team, 2022), using the packages ExpDes.pt (Ferreira et al., 2021), ggplot2 (Wickham, 2016) and candisc (Friendly and Fox, 2024).

## 3 Results and discussion

### 3.1 Nodular analysis

Analysis of variance revealed a significant effect of treatments on the number of nodules per plant (NNP) and nodule dry mass per plant (NDM), demonstrating that inoculation, co-inoculation, and re-inoculation techniques directly influenced the symbiotic activity in common beans. These results provide essential mechanistic validation for the efficiency of the applied biological treatments.

**Figure 2:**
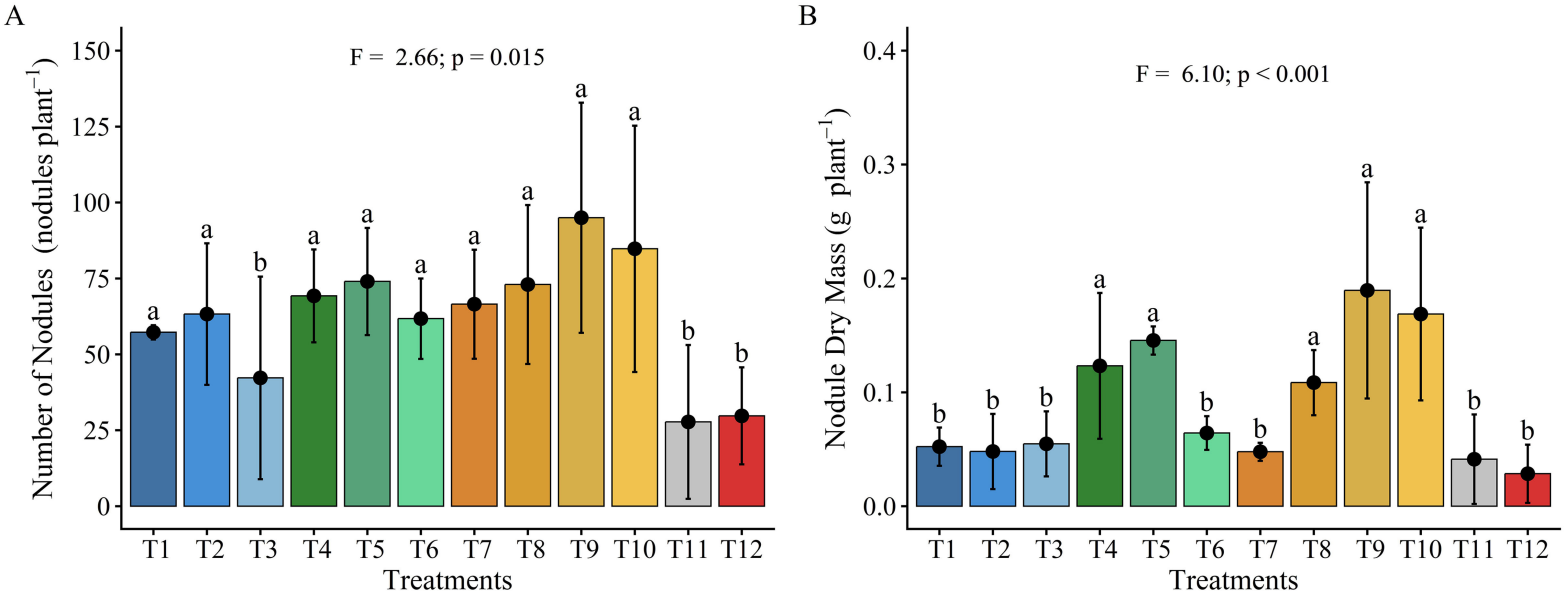
Effects of inoculation techniques, co-inoculation, and re-inoculation with *R. tropici* and *A. brasilense*, and mineral fertilization on common bean nodulation variables: number of nodules per plant **(A)** and nodule dry mass per plant **(B)**. T1, Seed inoculation with *R. tropici*; T2, In-furrow inoculation with *R. tropici*; T3, Top-dressing inoculation with *R. tropici*; T4, Seed co-inoculation (*R. tropici* + *A. brasilense*); T5, In-furrow co-inoculation (*R. tropici* + *A. brasilense*); T6, Top-dressing co-inoculation (*R. tropici* + *A. brasilense*); T7, T1 + subsequent top-dressing re-inoculation; T8, T2 + subsequent top-dressing re-inoculation; T9, T4 + subsequent top-dressing re-inoculation; T10, T5 + subsequent top-dressing re-inoculation; T11, Absolute control (no N source); T12, Mineral N fertilization at sowing and top-dressing (100 kg N ha^-1^). Points represent the means and vertical bars indicate the standard deviation. Distinct letters indicate significant differences among treatments according to the Scott-Knott test (p < 0.05).

Regarding NNP (**Figure 2A**), treatments involving the co-inoculation of *R. tropici* and *A. brasilense* (notably T4, T5, T9, and T10) showed the highest mean nodule counts. T4 (seed co-inoculation), T5 (in-furrow co-inoculation), T9 (seed co-inoculation + top-dress re-inoculation), and T10 (in-furrow co-inoculation + top-dress re-inoculation) presented means of 69, 74, 95, and 85 nodules per plant, respectively. This performance can be attributed to the synergistic effect of co-inoculation, where *A. brasilense* acts as PGPB by releasing phytohormones that modify root architecture. In addition to promoting overall root growth, this morphological alteration enhances rhizobium colonization, increasing the number of infection sites and nodule formation (Fiori et al., 2021).

These results surpass those observed by Ribeiro et al. (2024), who, despite using the same cultivar during the winter crop and combining co-inoculation of the same microorganisms with molybdenum and cobalt application, obtained a lower average of 20.4 nodules per plant. It is important to note that, in both this study and that of Ribeiro et al. (2024), only nodules with a diameter equal to or greater than 1.0 mm were counted. Conversely, Steiner et al. (2019) reported higher values when evaluating the cv. Pérola with the same combination of microorganisms, finding 45.4 nodules in the spring-summer and up to 166.5 in the summer-autumn. However, the authors did not specify a minimum diameter criterion for nodule counting, which may have contributed to the higher observed values. Several studies have highlighted the positive effect of rhizobia and azospirilla co-inoculation on promoting nodulation in different crops. Consistent results have been observed in other legumes, such as chickpea (Sharma et al., 2019), as well as in common beans (Bettiol et al., 2021).

As for NDM (**Figure 2B**), a similar trend was observed; treatments T4, T5, T9, and T10 stood out with the highest averages, ranging from 0.12 g plant^-^¹ (T4) to 0.19 g plant^-^¹ (T9), which, along with T8 (0.11 g plant^-^¹), were significantly superior to the others. This indicates that, beyond the higher quantity, the nodules exhibited greater biomass and development, signaling high symbiotic activity. This suggests that re-inoculation throughout the cycle can maintain active nodulation and recolonize active root sites even in more advanced stages, favoring symbiotic nitrogen supply, especially in short-cycle crops like common beans. Sousa et al. (2022b) obtained similar results when applying *R. tropici* to the seed with top-dress reinforcement, reaching approximately 0.20 g plant^-^¹ of nodule dry mass, corroborating the viability of this practice.

The efficacy of in-furrow co-inoculation is explained by the fact that this technique favors the permanence and direct contact of microorganisms with the root for a longer period, enhancing symbiosis. This application method was responsible for significant increments in both NNP and NDM, evidencing its role in promoting more efficient nodulation. As pointed out by (Ogutcu et al., 2008), nodule dry mass is one of the primary indicators of symbiotic effectiveness, being directly related to improved nitrogen nutrition and plant development. Clarifying that root colonization success and symbiotic establishment were directly confirmed by the significant increases in nodule number and nodule dry mass, which act as primary biological indicators of successful endophytic infection.

On the other hand, the remaining treatments presented the lowest NDM values, indicating low symbiotic efficiency. Notably, T12 (mineral nitrogen fertilization) and T11 (control without any N source) presented mean values of 0.03 and 0.04 g plant^-^¹, respectively. These data highlight a crucial point, the starter nitrogen dose 16 kg N ha^-^¹ applied at sowing in the biological treatments did not mask the treatment effects nor inhibit nodulation, successfully providing initial nutritional support without compromising symbiosis. Conversely, the results reinforce the hypothesis that high mineral nitrogen supply such as the 100 kg N ha^-^¹ in T12 can inhibit nodule formation and accelerate senescence, while the total absence of N limits both growth and symbiosis (Lepetit and Brouquisse, 2023; Ma et al., 2025). At the molecular level, high soil nitrate can trigger the production of CLE peptides, which repress the activation of nodulation-related genes, revealing a regulatory system through which the plant adjusts nodule formation according to the availability of nitrogen components in the soil (Lebedeva et al., 2020).

### 3.2 Morphological analyses

**Figure 3:**
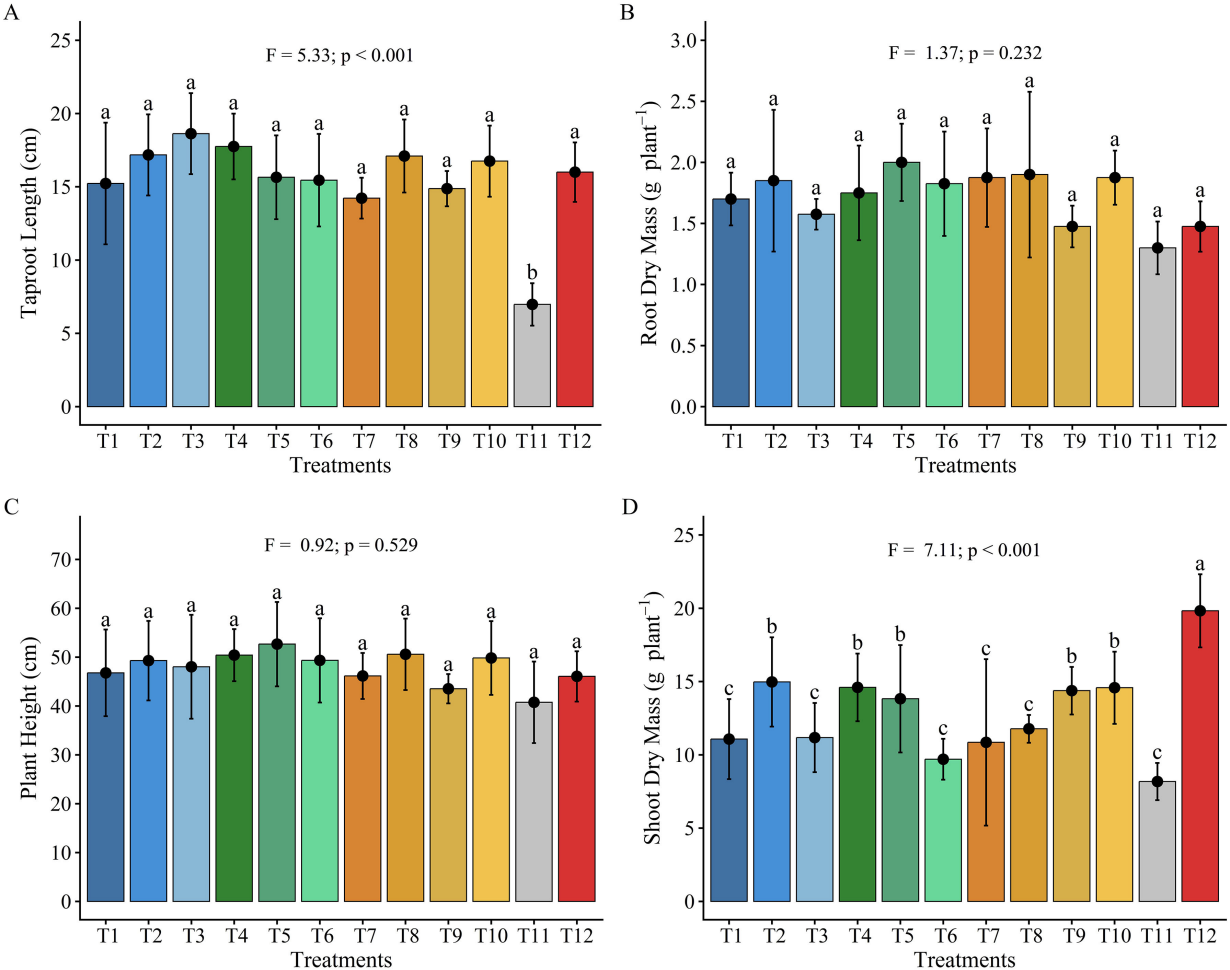
Effects of inoculation techniques, co-inoculation, and re-inoculation with *R. tropici* and *A. brasilense*, and mineral fertilization on common bean morphological traits: taproot length **(A)**, root dry mass **(B)**, plant height **(C)**, and shoot dry mass **(D)**. T1, Seed inoculation with *R. tropici*; T2, In-furrow inoculation with *R. tropici*; T3, Top-dressing inoculation with *R. tropici*; T4, Seed co-inoculation (*R. tropici* + *A. brasilense*); T5, In-furrow co-inoculation (*R. tropici* + *A. brasilense*); T6, Top-dressing co-inoculation (*R. tropici* + *A. brasilense*); T7, T1 + subsequent top-dressing re-inoculation; T8, T2 + subsequent top-dressing re-inoculation; T9, T4 + subsequent top-dressing re-inoculation; T10, T5 + subsequent top-dressing re-inoculation; T11, Absolute control (no N source); T12, Mineral N fertilization at sowing and top-dressing (100 kg N ha^-1^). Points represent the means and vertical bars indicate the standard deviation. Distinct letters indicate significant differences among treatments according to the Scott-Knott test (p < 0.05).

The analysis of variance revealed significant differences among treatments regarding taproot length (**Figure 3A**). Treatments T1 to T12, with the exception of T11, formed a statistically superior homogeneous group, with mean values ranging from 14.2 to 18.6 cm. Although they presented numerical variations, these treatments did not differ statistically from each other regarding TRL. Only T11 (control without any N source) showed significantly lower performance, with a mean of 7 cm, highlighting the limitation in promoting root development in the absence of any N source. These results demonstrate that both inoculation and co-inoculation with *R. tropici* and *A. brasilense*, with or without re-inoculation, positively stimulated the growth of the common bean taproot.

Similar results were reported by Silva et al. (2025) in bean cultivars subjected to co-inoculation. In the spring-summer crop, these authors observed that co-inoculation resulted in a TRL of 28.9 cm, an increase of 10.9% compared to inoculation with *Rhizobium* alone (26 cm). In the following season, the authors also observed greater root development with soil application of co-inoculation (27.3 cm) compared to seed application (22.3 cm). Almeida et al. (2022) also highlighted the positive influence of co-inoculation and re-inoculation on root growth of cultivars BRS Estilo and BRS Esteio, confirming that co-inoculation with *A. brasilense* resulted in increased root length compared to those without co-inoculation.

The performance of the mineral fertilization treatment (T12), equivalent to that of biological treatments, reinforces the potential of inoculation techniques as a viable and sustainable tool for integrated nutrient management. In contrast, T11, without any nitrogen source, presented the lowest TRL, evidencing significant losses in the absence of any form of N supply.

For root dry mass, no significant statistical difference was detected among treatments (**Figure 3B**). This suggests that, despite the variation in TRL of T11, root biomass accumulation was similar among treatments. This homogeneity may be related to the plant development stage at the time of sampling, sampling issues, or the limitation of other edaphic factors, such as soil moisture or structure (Helliwell et al., 2017), which may have influenced root development uniformly.

Plant height also showed no significant differences among treatments (**Figure 3C**), indicating that bean height growth was not influenced by different inoculation techniques. Means ranged numerically from 40.8 cm (T11 – control without any N source) to 52.7 cm (T5 – in-furrow co-inoculation), without these variations constituting significant differences. It can be inferred that, at this phenological stage, biomass accumulation and root development may reflect the effects of inoculation and fertilization more clearly, unlike PH, which does not demonstrate this relationship evidently. Furthermore, part of this stability in height among treatments can be attributed to the predominance of genetic factors in the expression of this trait.

Shoot dry mass showed significant differences among treatments (**Figure 3D**), being a highly sensitive characteristic to the tested management practices. The treatment with mineral fertilization (T12) showed the highest shoot biomass accumulation (19.8 g plant^-1^), differing statistically from the others. This result reinforces the efficiency of direct N supply on vegetative shoot development, as this nutrient stimulates leaf area expansion, raises photosynthetic rates, and favors biomass accumulation. Moreover, its greater availability in the soil, provided by fertilization, increases uptake by roots and N content in the shoot, which translates into greater dry mass production in common bean (Marschner, 2011). A similar result was observed by Ribeiro et al. (2024), who reported higher mean values of SDM in treatments with mineral fertilization and re-inoculation with co-inoculation of *R. tropici* + *A. brasilense* + Mo/Co.

However, some biological treatments also performed well, such as T2 (in-furrow inoculation) with 15 g plant^-1^, T4 (seed co-inoculation) with 14.6 g plant^-1^, T10 (in-furrow co-inoculation with top-dressing re-inoculation) with 14.6 g plant^-1^, T9 (seed co-inoculation with top-dressing re-inoculation) with 14.4 g plant^-1^, and T5 (in-furrow co-inoculation) with 13.8 g plant^-1^, which formed a second statistically superior group. These results indicate that inoculation and co-inoculation, both via seed and in-furrow, especially when associated with top-dressing re-inoculation, can be efficient strategies to promote shoot dry matter accumulation, offering a promising and sustainable alternative to reduce the reliance on traditional nitrogen fertilization.

According to Steiner et al. (2019), common bean plants co-inoculadas with *R. tropici* and *A. brasilense* showed increases of 96% in leaf area, 43% in root volume, and 30% in total dry matter compared to non-inoculated plants, even under water stress. These positive effects can be attributed to the synergism between bacteria, as pointed out by (Messias et al., 2023b), who highlight the contribution of biological N fixation, phytohormone production, and phosphate solubilization. To properly address these synergistic benefits, it is essential to differentiate their mechanisms, while *R. tropici* acts primarily through direct nitrogen supply via BNF, *A. brasilense* functions predominantly as a PGPB. It produces phytohormones, particularly indole-3-acetic acid (IAA), which stimulate cell elongation and significantly alter root architecture. This expanded root system maximizes water and nutrient interception and can promote plant functional resistance to biotic and abiotic stresses, resulting in greater growth and vigor, as observed by (Bulgarelli et al., 2013).

### 3.3 Physiological analyses

The analysis of variance revealed significant differences among treatments regarding chlorophyll content (CC), net photosynthetic rate (A), and internal CO_2_ concentration (Ci), while for transpiration rate (E), no statistical difference was observed (p ≥ 0.05). These results evidence that inoculation and co-inoculation strategies mainly interfere with photosynthetic processes and photosynthetic pigment accumulation, with a lesser effect on the physiological parameter related to plant transpiration under the experimental conditions.

**Figure 4:**
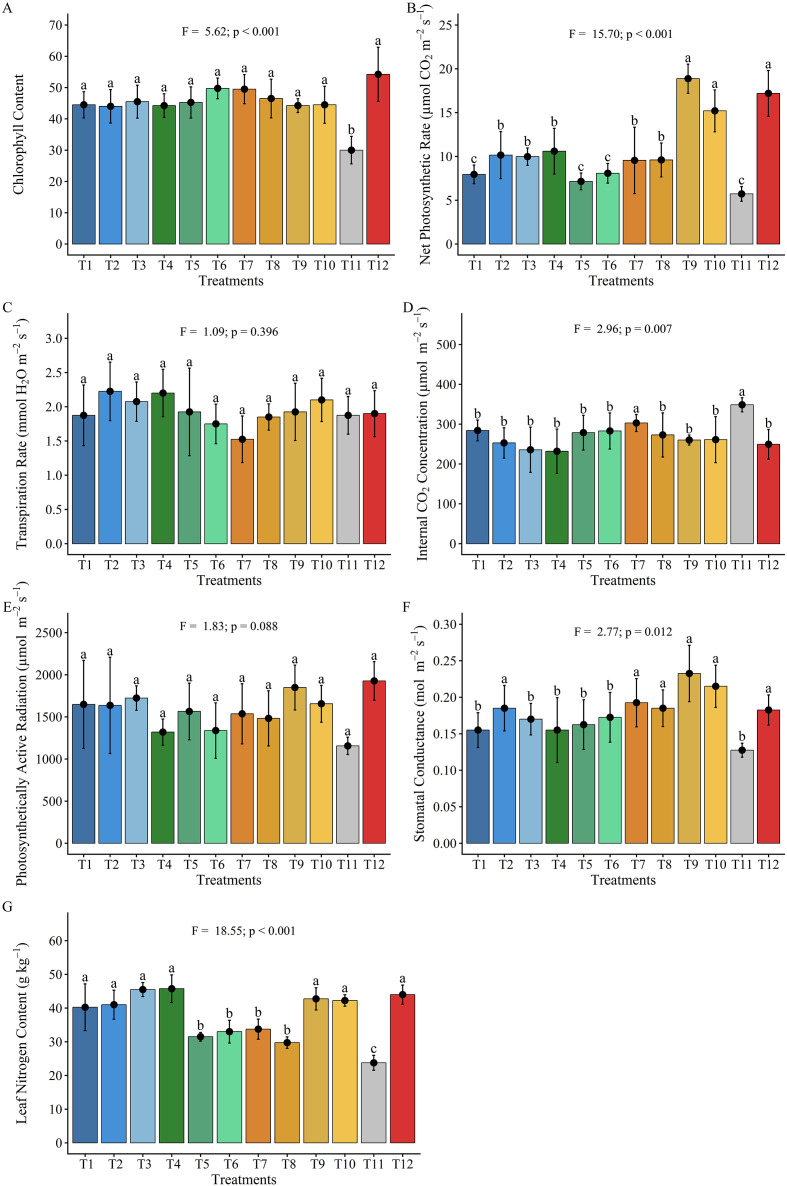
Effects of inoculation techniques, co-inoculation, and re-inoculation with *R. tropici* and *A. brasilense*, and mineral fertilization on common bean physiological traits: chlorophyll content **(A)**, net photosynthetic rate **(B)**, transpiration rate **(C)**, internal CO_2_ concentration **(D)**, photosynthetically active radiation **(E)**, stomatal conductance **(F)**, and leaf nitrogen content **(G)**. T1, Seed inoculation with *R. tropici*; T2, In-furrow inoculation with *R. tropici*; T3, Top-dressing inoculation with *R. tropici*; T4, Seed co-inoculation (*R. tropici* + *A. brasilense*); T5, In-furrow co-inoculation (*R. tropici* + *A. brasilense*); T6, Top-dressing co-inoculation (*R. tropici* + *A. brasilense*); T7, T1 + subsequent top-dressing re-inoculation; T8, T2 + subsequent top-dressing re-inoculation; T9, T4 + subsequent top-dressing re-inoculation; T10, T5 + subsequent top-dressing re-inoculation; T11, Absolute control (no N source); T12, Mineral N fertilization at sowing and top-dressing (100 kg N ha^-1^). Points represent the means and vertical bars indicate the standard deviation. Distinct letters indicate significant differences among treatments according to the Scott-Knott test (p < 0.05).

Regarding CC, a significant statistical difference was observed among treatments (**Figure 4A**). All biological treatments presented levels significantly higher than the control without nitrogen supply (T11), which obtained the lowest value (approx. 30, dimensionless). This reinforces the fundamental role of N in the synthesis of leaf pigments, especially chlorophyll, which is essential for photosynthesis (Fathi, 2022). Treatments T1 to T10 showed mean values ranging between 44 and 50, statistically similar to each other, evidencing the efficiency of inoculation and co-inoculation techniques with diazotrophic bacteria in promoting chlorophyll accumulation. The mineral nitrogen fertilization treatment (T12), with a mean value of 54, also did not differ statistically from biological treatments, suggesting that symbiotic nitrogen supply can effectively meet part of the crop’s nutritional requirements.

This result reinforces the relevance of nitrogen for plant metabolism, as this element is directly involved in the constitution of essential compounds such as amino acids, nucleic acids, proteins, and photosynthetic pigments like chlorophyll (Mendoza-Tafolla et al., 2019). Furthermore, studies demonstrate that N availability directly influences chlorophyll accumulation in leaves, positively impacting chlorophyll *a* and *b* levels (Muhammad et al., 2022). Similar results were observed by (Steiner et al., 2019), who reported an increase in the relative chlorophyll index in common bean plants inoculated and co-inoculated with *R. tropici* and *A. brasilense*, both under non-stress and moderate stress conditions, confirming the efficacy of these specific microorganisms in promoting leaf health and enhancing the photosynthetic potential of legumes.

Net A showed significant differences among treatments (**Figure 4B**). Treatments T9 (seed co-inoculation with top-dressing re-inoculation), T12 (mineral fertilization), and T10 (in-furrow co-inoculation with subsequent top-dressing re-inoculation) obtained the highest means, with 18.9, 17.2, and 15.2 µmol CO_2_ m^-2^ s^-1^, respectively, with no statistical difference among them. This result suggests a possible synergistic effect between *R. tropici* and *A. brasilense*, promoting greater efficiency in atmospheric CO_2_ use under field conditions controlled for diurnal variations. Similar results were observed by (Ayalew et al., 2022), who reported that inoculation with *Bradyrhizobium* significantly increased net photosynthesis across different growing seasons compared to the uninoculated control in cowpea (*Vigna unguiculata*), highlighting the broad potential of symbiotic bacteria in improving the photosynthetic activity of legumes.

For E, no significant differences were observed among treatments (**Figure 4C**), values ranged around 1.5 to 2.2 mmol H_2_O m^-2^ s^-1^, indicating that the presence or absence of bacteria did not directly influence transpiration fluxes through leaves. This behavior can be attributed to adequate water availability throughout the crop cycle (**Figure 1**). Under conditions without water restriction, plants can express their maximum transpiration potential, maintaining stomatal opening for longer periods, which favors both CO_2_ uptake and water absorption. Furthermore, maintaining active transpiration is a fundamental physiological process for nutrient transport via mass flow, such as nitrogen and potassium, promoting adequate plant nutrition.

Regarding Ci, significant differences were observed among treatments (**Figure 4D**). The highest values were recorded in the control without N (T11), with 348 µmol m^-2^ s^-1^, and in the seed inoculation treatment with top-dressing re-inoculation (T7), with 303 µmol m^-2^ s^-1^, both superior to the others. To accurately interpret this Ci accumulation, it is crucial to differentiate between stomatal and biochemical limitations. In T11, considering that stomatal conductance was active, the high Ci coupled with the lowest photosynthetic rate indicates a severe biochemical limitation rather than a stomatal restriction. The lack of nitrogen supply compromises the synthesis and activity of the Ribulose-1,5-bisphosphate carboxylase/oxygenase (Rubisco) enzyme. Consequently, the CO_2_ enters the substomatal cavity but is not efficiently carboxylated, leading to internal accumulation. Generally, lower Ci values observed in the other treatments (ranging from 232 to 284 µmol m^-2^ s^-1^) indicate greater carbon fixation and photosynthetic efficiency, as reduced internal CO_2_ concentrations are directly associated with higher Rubisco activity and efficient carboxylation.

Thus, results demonstrate that both inoculation and co-inoculation, with or without re-inoculation, promoted greater CO_2_ assimilation, reflecting better photosynthetic performance in bean plants compared to the control without N. Mineral fertilization (T12) also behaved similarly to biological treatments, indicating that the integration of bioinputs was efficient enough to partially replace nitrogen fertilization.

For PAR, no significant differences were observed among treatments (**Figure 4E**). PAR varied from 1156.3 µmol m^-2^ s^-1^ (T11 - control without N) to 1927.3 µmol m^-2^ s^-1^ (T12 - mineral N fertilization), but all treatments were grouped within the same comparison level. The absence of statistical differences in PAR suggests that inoculation, co-inoculation, and re-inoculation techniques did not significantly influence the amount of radiation available for photosynthetic processes. It is noteworthy that PAR is strongly determined by environmental factors, such as luminosity and climatic conditions (Proutsos et al., 2022), being, therefore, less directly affected by biological management practices like microorganism inoculation. Although treatment T12 presented the numerically highest PAR value and control T11 the lowest, indicating that nitrogen supply, whether by mineral fertilization or biological fixation, did not expressively alter the plants’ capacity to intercept light for photosynthesis. This result reinforces that BNF management impacts physiological variables related to internal plant metabolism more directly, such as photosynthesis (Jumrani et al., 2023), and biomass production (Lele and Kebede, 2022), while radiation interception depends mostly on environmental factors (Duan et al., 2025).

When analyzing gs, bean plants were significantly influenced by different inoculation, co-inoculation, and re-inoculation techniques (**Figure 4F**). Treatments T9, T10, T7, T2, T8, and T12 showed the highest mean gs, ranging from 0.23 to 0.18 mol m^-2^ s^-1^, with T9 (seed co-inoculation with top-dressing re-inoculation) standing out, followed by T10 and T7, which also involved re-inoculation. The absolute control (T11), without nitrogen supply, recorded the lowest value (0.13 mol m^-2^ s^-1^), indicating physiological limitation. Stomatal conductance is directly related to stomatal opening, regulating CO_2_ flux and, consequently, the photosynthetic rate. Higher gs in re-inoculation treatments can be attributed to greater nitrogen availability via biological fixation, favoring plant metabolism. These findings align with (Arruda et al., 2023), who observed higher gs and transpiration in common bean cultivated in clayey soil with inoculation, while non-inoculated plants showed low gs, evidencing stomatal closure in response to osmotic stress.

Leaf nitrogen content was significantly influenced by different inoculation, co-inoculation, re-inoculation, and mineral fertilization strategies (**Figure 4G**). Treatments T4, T3, T12, T9, T10, T2, and T1 were classified with the highest LNC levels, ranging between 40 and 46 g kg^-1^, which did not differ statistically from each other. This demonstrates that top-dressing application of *R. tropici* (T3), co-inoculation with re-inoculation (T9 and T10), and conventional nitrogen fertilization (T12) promoted maximum foliar N assimilation. Treatments T7, T6, T5, and T8 formed a group with intermediate levels (29 to 34 g kg^-1^ N). The control T11 presented the lowest value (24 g kg^-1^ N). LNC is a key indicator of plant nitrogen nutrition and is directly related to BNF efficiency or mineral fertilizer supply. The strong positive relationship observed between the highest LNC values and the highest A in treatments like T9 and T12 confirms that robust nitrogen nutrition is the primary driver for maintaining high carboxylation efficiency.

Treatments including re-inoculation, whether with *R. tropici* alone or co-inoculated with *A. brasilense*, presented the highest N levels, indicating greater symbiotic efficiency and better supply of assimilable nitrogen to plants. Re-inoculation, by reinforcing symbiotic activity throughout the crop cycle, contributed to maintaining N supply during more demanding development phases. Furthermore, N levels found in the most efficient treatments were within the range considered adequate for common bean at flowering (30 to 50 g kg^-1^), as proposed by (Ambrosano et al., 1997), evidencing that well-managed biological practices can meet the plant’s nutritional demand. This BNF efficacy was also highlighted by (Assegid and Abera, 2023) who observed greater N accumulation in inoculated plants. The equivalence between the best biological treatments and conventional nitrogen fertilization reinforces the potential of bioinputs as an alternative to chemical fertilizers, contributing to more sustainable systems.

Conversely, treatments with intermediate LNC levels suggest that single inoculation or lack of symbiotic reinforcement throughout the cycle may limit BNF efficacy. This may be related to lower root colonization, competition with native microorganisms, or decreased nodular activity at advanced stages, directly impacting nutrition and plant biomass. The absolute control presented the lowest leaf content (24 g kg^-1^), confirming nutritional deficiency. This result corroborates findings by (Wekesa et al., 2021), who observed lower nitrogen and chlorophyll content in non-inoculated plants, with negative reflections on leaf color and biomass production.

To further strengthen these physiological interpretations, a simple Pearson correlation analysis confirmed a significant positive relationship between leaf nitrogen content and the net photosynthetic rate (r = 0.56, p < 0.05). Considering the multifactorial nature of gas exchange dynamics under field conditions, this direct correlation mathematically validates that the enhanced nutritional status provided by the symbiotic interactions effectively supported the biochemical demands of the photosynthetic apparatus, likely driving Rubisco synthesis and activity.

### 3.4 Agronomic analyses

**Figure 5:**
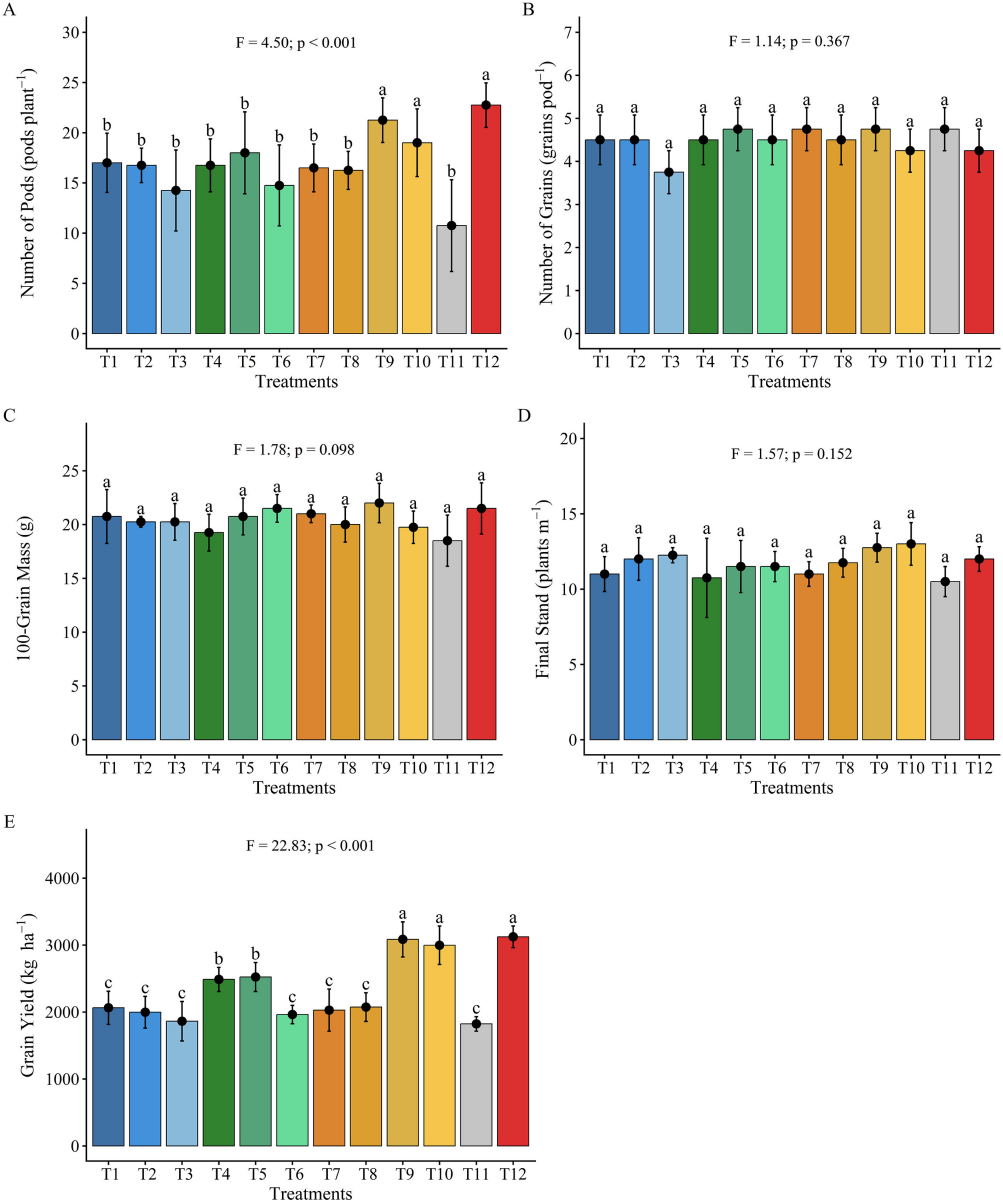
Effects of inoculation techniques, co-inoculation with *R. tropici* + *A. brasilense*, and mineral fertilization on common bean agronomic traits: number of pods per plant **(A)**, number of grains per pod **(B)**, 100-grain mass **(C)**, final stand **(D)**, and grain yield **(E)**. T1, Seed inoculation with *R. tropici*; T2, In-furrow inoculation with *R. tropici*; T3, Top-dressing inoculation with *R. tropici*; T4, Seed co-inoculation (*R. tropici* + *A. brasilense*); T5, In-furrow co-inoculation (*R. tropici* + *A. brasilense*); T6, Top-dressing co-inoculation (*R. tropici* + *A. brasilense*); T7, T1 + subsequent top-dressing re-inoculation; T8, T2 + subsequent top-dressing re-inoculation; T9, T4 + subsequent top-dressing re-inoculation; T10, T5 + subsequent top-dressing re-inoculation; T11, Absolute control (no N source); T12, Mineral N fertilization at sowing and top-dressing (100 kg N ha^-1^). Points represent the means and vertical bars indicate the standard deviation. Distinct letters indicate significant differences among treatments according to the Scott-Knott test (p < 0.05).

The number of pods per plant was significantly influenced by the different inoculation strategies evaluated, evidencing that biological nitrogen management directly impacts this productive component of the common bean (**Figure 5A**). Treatments T12 (mineral nitrogen fertilization), T9 (seed co-inoculation with re-inoculation), and T10 (in-furrow co-inoculation with re-inoculation) showed the highest NPP values, with means ranging between 19 and 23 pods per plant. These results reinforce the efficacy of co-inoculation associated with re-inoculation and mineral fertilization, indicating that these practices favor the crop’s reproductive development, likely through improvements in symbiotic nitrogen supply during critical moments of the crop cycle. Similar results were reported by (Reis et al., 2023), in which treatments with *R. tropici* + *A. brasilense* and *R. tropici* alone increased the number of pods per plant by 51% and 47%, respectively, compared to the non-inoculated control. Likewise, Bravo et al. (2025) reported that the highest NPP values were obtained under mineral fertilization, followed closely by the co-inoculated treatment applied via seed and re-inoculated through top-dressing, reinforcing the potential of biological inoculation strategies to enhance reproductive performance and mitigate the effects of nitrogen limitation on bean yield. Treatments T1, T2, T3, T4, T5, T6, T7, and T8 showed intermediate performance, while the lowest value was observed in the absolute control treatment (T11).

The low pod productivity observed in the control (T11), with an average of only 11 pods per plant, highlights the importance of adequate nitrogen supply, whether via fertilization or symbiosis, for the crop’s productive success. Co-inoculation, especially in association with symbiotic reinforcement throughout the cycle, seems to enhance root colonization and nutrient availability. Thus, it is worth noting that the NPP is considered one of the main determinants of bean productivity (Fageria et al., 2014), being influenced not only by genetic factors but also by environmental conditions and adopted management. The data obtained in this study evidence that well-planned inoculation techniques, especially co-inoculation with re-inoculation, can significantly contribute to the increment of crop yield components.

The number of grains per pod, 100-grain mass, and final stand did not present significant statistical differences among treatments (**Figures 5B–D**). These results indicate that, although strategies involving co-inoculation and re-inoculation promoted gains in the NPP, these management practices did not directly influence the other productive components at a statistically detectable level.

Grain yield showed significant statistical differences among treatments (**Figure 5E**), evidencing that different inoculation, co-inoculation, and re-inoculation techniques impacted bean productive performance distinctly. Treatments T9, T10, and T12 stood out with the highest yields, reaching 3,085 kg ha^-1^, 2,998 kg ha^-1^, and 3,124 kg ha^-1^, respectively. These results indicate that co-inoculation of *R. tropici* + *A. brasilense* via seed (T9) or in-furrow (T10), associated with top-dressing re-inoculation, was as efficient as mineral N fertilization (T12), highlighting the potential of biological strategies to maximize crop productivity.

These findings are corroborated by (Bettiol et al., 2021), who observed a yield of 3,295 kg ha^-1^ in common bean with the combination of *R. tropici* inoculation, foliar application of *A. brasilense*, and top-dressing nitrogen fertilization with 90 kg N ha^-1^, representing a 27% increase compared to the control. Pastor-Bueis et al. (2020) also reported an average increase of nearly 1,000 kg ha^-1^ (over 40%) in productivity with single or intercropped inoculation of *Rhizobium*, while Jalal et al. (2021) verified an increment of up to 54.3% in yield with co-inoculation of *R. tropici* + *Bacillus subtilis*, regardless of Zn application. These studies reinforce the agronomic value of associations between rhizobia and PGPB, such as *A. brasilense*, whose action goes beyond simple N supply, including phytohormone production and improved nutrient absorption (Hungria et al., 2013; Puente et al., 2018).

In contrast, treatments involving only *R. tropici* inoculation (T1, T2, T3) or inoculation combined with re-inoculation (T7 and T8) showed the lowest yields, ranging between 1,863 and 2,075 kg ha^-1^. These data show that simple inoculation or isolated re-inoculation is not sufficient to guarantee high productive levels, especially in the absence of co-inoculation with PGPB. This is consistent with results by (Oliveira et al., 2025), who reported yields between 978 and 2,022 kg ha^-1^ in soils with different types of inoculation without nitrogen fertilizer.

Treatments T4 and T5, involving co-inoculation without re-inoculation, presented intermediate yields, between 2,487 and 2,524 kg ha^-1^, indicating that even in the absence of re-inoculation, the presence of *A. brasilense* already confers relevant agronomic benefits, aligning with results by (Steiner et al., 2020), who observed improvement in grain yield and production components with inoculation of symbiotic and non-symbiotic bacteria. Furthermore, the absolute control (T11), without any nitrogen source, showed the lowest grain yield (1,823 kg ha^-1^), evidencing the essentiality of N supply, whether via mineral fertilization or symbiosis, for adequate crop productive performance when aiming for yields superior to 2,500 kg ha^-1^.

In general, results indicate that co-inoculation (via seed or soil) with top-dressing re-inoculation of *R. tropici* + *A. brasilense* is a technology of high agronomic efficiency, capable of significantly increasing crop yield, reaching values superior to 3,000 kg ha^-1^. This practice represents a sustainable and economically viable alternative, with the potential to reduce dependence on nitrogen fertilizers. Considering the current high costs of urea and other synthetic nitrogen sources, achieving statistical equivalence in yield with T12 using only inoculants and a small starter N dose demonstrates a substantial economic advantage for the farmer, promoting more resilient and environmentally responsible production systems.

### 3.5 Agronomic efficiency and economic analysis

The evaluation of Nitrogen Use Efficiency (NUE) metrics underscored the profound agronomic advantage of the integrated biological management. By calculating the Agronomic Efficiency of Nitrogen (AEN) defined as the increase in grain yield per unit of applied N relative to the absolute control (T11) distinct physiological performances were revealed. The conventional mineral treatment (T12), which received a full dose of 100 kg N ha^-^¹, exhibited an AEN of 13.0 kg of grain per kg of applied N. In striking contrast, the co-inoculated and re-inoculated treatments (T9 and T10), relying exclusively on a minimal starter dose of 16 kg N ha^-^¹, achieved an exceptional average AEN of 76.1 kg of grain per kg of applied N.

Crucially, this massive increment in agronomic efficiency is not merely a mathematical artifact resulting from a reduced synthetic N input, but rather the direct physiological outcome of the microbial symbiosis. The synergistic association between *R. tropici* and *A. brasilense* compensated for the lack of chemical nitrogen top-dressing, acting as a biological multiplier of fertilizer efficiency. While the starter N dose ensured seedling establishment prior to full nodule maturation, the subsequent biological nitrogen fixation by *R. tropici* and the enhanced root foraging capacity promoted by *A. brasilense* provided the necessary physiological sustainment for late-stage grain filling. Ultimately, this integrated strategy maximizes the conversion of minimal baseline nutrients into harvestable grain biomass.

Economic analysis of the treatments is essential to assess the practical viability of biological technologies in common bean crops, especially when compared to traditional full mineral nitrogen fertilization. A cost simulation was performed considering the financial performance of three management strategies: full mineral nitrogen fertilization (T12), and the integrated biological treatments involving co-inoculation plus top-dressing re-inoculation via seed (T9) or in-furrow (T10). The economic assessment was based on average input prices in the Brazilian market (converted to US Dollars considering an exchange rate of US$ 1.00 = R$ 6.00, as of November 2024). Mineral nitrogen as urea, 46% N was valued at US$ 599.47 t^-^¹, *R. tropici* at US$ 6.67 dose^-^¹, *A. brasilense* at US$ 5.83 dose^-^¹, and the tractor operation cost for top-dressing biological application was estimated at US$ 15.00 ha^-^¹.

The inputs and operational cost structures revealed a clear economic advantage for the integrated biological route. The standard mineral treatment (T12), which received a total dose of 100 kg N ha^-^¹ equivalent to 217.4 kg ha^-^¹ of urea, resulted in a total management investment of US$ 130.32 ha^-^¹. Conversely, the biological management strategies (T9 and T10), which combined a starter nitrogen dose at sowing 16 kg N ha^-^¹, equivalent to 34.8 kg ha^-^¹ of urea, with double co-inoculation at sowing and double re-inoculation top-dressed at V4, plus the tractor operational cost, incurred a total expenditure of US$ 60.86 ha^-^¹. This represents a 53.3% reduction in direct investment per hectare for nitrogen management, establishing a clear cost-saving advantage for the biological strategies.

When evaluating the cost-benefit ratio per 60-kg bag produced (US$ bag^-^¹), which links the management investment directly to grain yield, the economic efficiency of the bioinputs becomes even more evident. The full mineral fertilization (T12) achieved a grain yield of 3,124 kg ha^-^¹ (52.1 bags ha^-^¹), resulting in a management cost of US$ 2.50 per bag produced. Strikingly, the biological treatments T9 and T10 achieved statistically equivalent yields, averaging 3,042 kg ha^-^¹ (50.7 bags ha^-^¹), which translated into a production cost of only US$ 1.20 per bag.

Furthermore, the efficiency of the co-inoculation of *R. tropici* and *A. brasilense* in common bean cultivation, compared to the use of nitrogen fertilizers, can also be confirmed in economic terms, as demonstrated by a recent study conducted by (Messias et al., 2023a). In this study, the average production cost was US$ 451 with the use of inoculation/co-inoculation, whereas the use of mineral nitrogen fertilizer resulted in a cost of US$ 499, representing a direct saving of approximately US$ 48 ha^-^¹, thus confirming the reduction in production costs and the increase in profitability of common bean cultivation. This advantage is associated with the low operational cost of inoculants, the partial or complete reduction in the need for urea, the lower logistical costs related to the acquisition and application of mineral N, and the improvement in nutrient uptake efficiency through BNF.

Despite the promising results observed for re-inoculation with *R. tropici* and *A. brasilense*, it is important to emphasize that this study was conducted in a single season and location. Therefore, although the data provide a solid foundation for understanding the physiological and agronomic effects of these techniques on common beans, the environment × treatment interactions still need to be validated across different edaphoclimatic conditions and agricultural years to consolidate these recommendations.

### 3.6 Canonical discriminant analysis

#### 3.6.1 Nodulation variables

**Figure 6:**
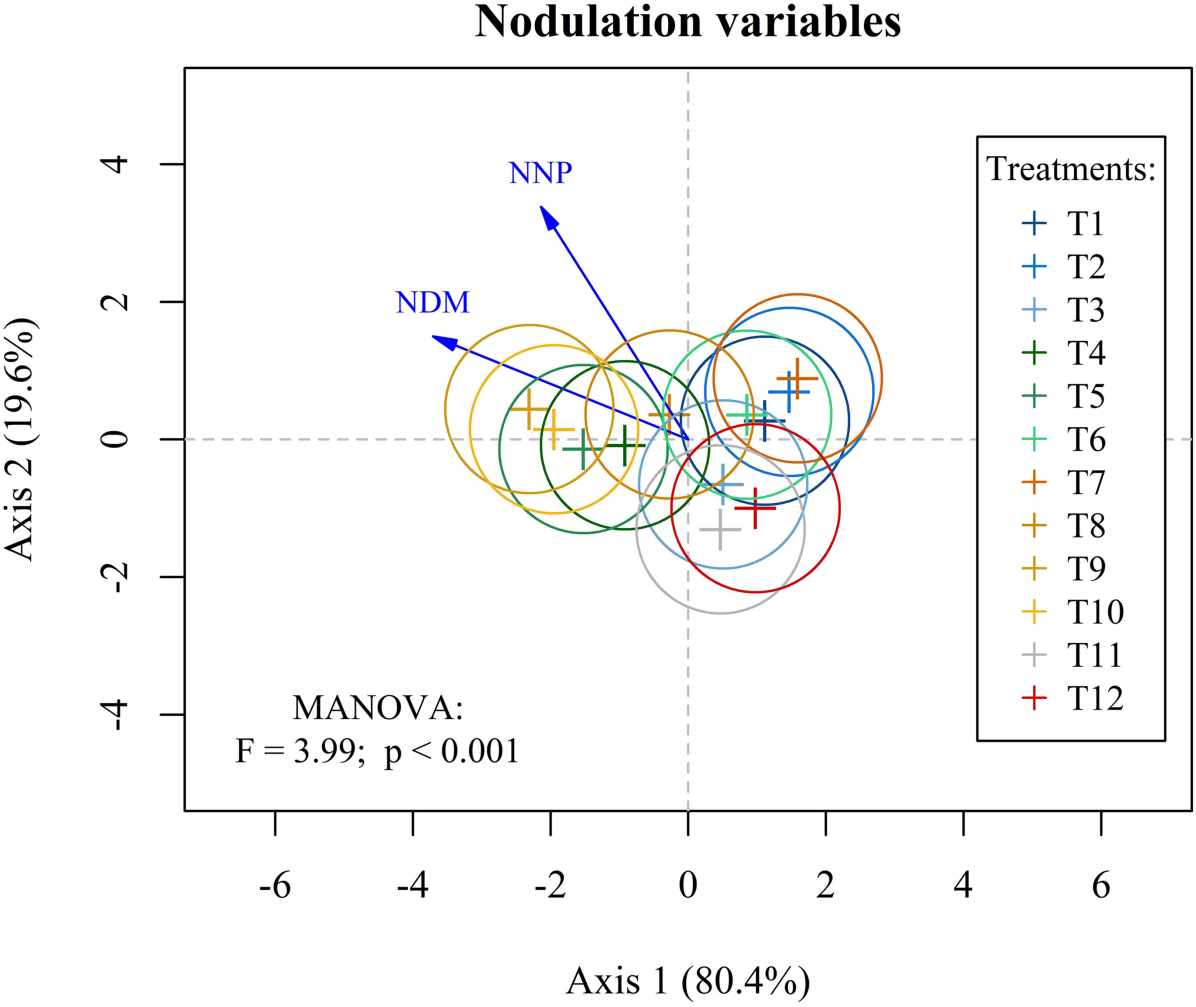
Canonical Discriminant Analysis of common bean in response to inoculation techniques, co-inoculation, re-inoculation, and mineral fertilization based on nodulation variables: number of nodules per plant (NNP) and nodule dry mass per plant (NDM). T1, Seed inoculation with *R. tropici*; T2, In-furrow inoculation with *R. tropici*; T3, Top-dressing inoculation with *R. tropici*; T4, Seed co-inoculation (*R. tropici* + *A. brasilense*); T5, In-furrow co-inoculation (*R. tropici* + *A. brasilense*); T6, Top-dressing co-inoculation (R. tropici + A. brasilense); T7, T1 + subsequent top-dressing re-inoculation; T8, T2 + subsequent top-dressing re-inoculation; T9, T4 + subsequent top-dressing re-inoculation; T10, T5 + subsequent top-dressing re-inoculation; T11, Absolute control (no N source); T12, Mineral N fertilization at sowing and top-dressing (100 kg N ha^-1^). The MANOVA results indicated a significant effect of treatments on morphophysiological variables (F = 3.98; p < 0.001), with confidence ellipses (95% level) representing group dispersion.

Canonical Discriminant Analysis allowed for the integration of the nodular responses of common beans into a multivariate approach, revealing clustering patterns among treatments based on the variables of number of nodules per plant and nodule dry mass per plant. The analysis was significant, and the first two canonical axes explained 100% of the variation, with 80.4% attributed to Axis 1 and 19.6% to Axis 2 (**Figure 6**).

The distribution of treatments in the canonical space indicated that NNP and NDM exhibited vectors directed toward the upper-left region of the plot, evidencing a positive correlation between them, albeit with slightly distinct orientations. Treatments positioned near these vectors, especially those involving the co-inoculation of *R. tropici* with *A. brasilense* associated with re-inoculation, tended to show superior nodulation performance. Notable among these are T9 (seed co-inoculation with re-inoculation) and T10 (in-furrow co-inoculation with re-inoculation), which were associated with the highest values for NNP and NDM. This multivariate clustering visually confirms the synergistic interaction described earlier, where structural root modifications promoted by the PGPB create an optimal environment for maximizing rhizobial infection sites.

Treatments with a central position in the plot showed a balanced response between nodule number and dry mass, suggesting functional maintenance of the symbiosis throughout the cycle, such as T8 (in-furrow inoculation of *R. tropici* with re-inoculation). In contrast, T11 (control without N) and T12 (mineral fertilization) were located further from the explanatory vectors, indicating poor nodular performance in the case of nitrogen fertilization, likely due to the inhibition of nodulation by the high mineral N supply (100 kg N ha^-^¹).

CDA complements the univariate analysis by providing an integrated view of symbiotic responses, highlighting qualitative differences among treatments. The results underscore co-inoculation, particularly when combined with re-inoculation, as a practice that favors both the quantity and the development of nodules. Thus, multivariate analysis reinforces the superiority of integrated biological practices, such as co-inoculation and top-dress re-inoculation, as highly effective components of an integrated nutrient management system, compared to isolated inoculation or the exclusive use of nitrogen fertilizers.

#### 3.6.2 Morphophysiological variables

**Figure 7:**
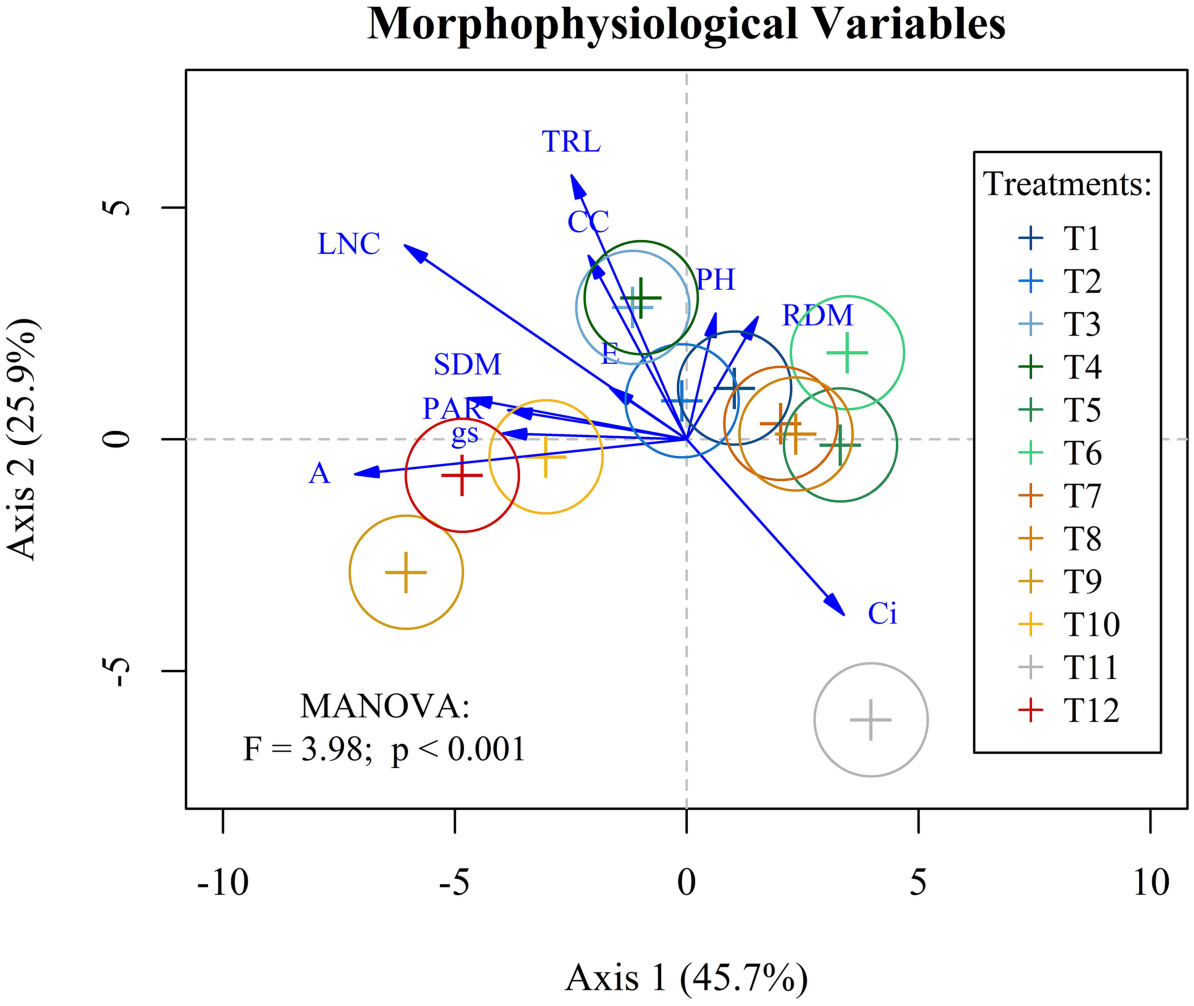
Canonical Discriminant Analysis (CDA) of common bean in response to inoculation techniques, co-inoculation, re-inoculation, and mineral fertilization based on morphophysiological variables: taproot length (TRL), plant height (PH), root dry mass (RDM), shoot dry mass (SDM), chlorophyll content (CC), net photosynthetic rate (A), transpiration rate (E), internal CO_2_ concentration (Ci), photosynthetically active radiation (PAR), stomatal conductance (gs), and leaf nitrogen content (LNC). T1, Seed inoculation with *R. tropici*; T2, In-furrow inoculation with *R. tropici*; T3, Top-dressing inoculation with *R. tropici*; T4, Seed co-inoculation (*R. tropici* + *A. brasilense*); T5, In-furrow co-inoculation (*R. tropici* + *A. brasilense*); T6, Top-dressing co-inoculation (R. tropici + A. brasilense); T7, T1 + subsequent top-dressing re-inoculation; T8, T2 + subsequent top-dressing re-inoculation; T9, T4 + subsequent top-dressing re-inoculation; T10, T5 + subsequent top-dressing re-inoculation; T11, Absolute control (no N source); T12, Mineral N fertilization at sowing and top-dressing (100 kg N ha^-1^). The MANOVA results indicated a significant effect of treatments on morphophysiological variables (F = 3.98; p < 0.001), with confidence ellipses (95% level) representing group dispersion.

The CDA evidenced the separation among treatments based on the morphophysiological variables of the common bean (**Figure 7**). The analysis was statistically significant, as indicated by MANOVA, and the first two canonical axes jointly explained 71.6% of the total variance among treatments, with 45.7% by Axis 1 and 25.9% by Axis 2. Although ANOVA revealed point differences in specific variables, CDA provided a multivariate view, highlighting the joint response pattern of plants to treatments. This type of approach is especially relevant in physiological studies, where variables are often interrelated and reflect coordinated responses of plant metabolism.

The projection of treatments in the canonical space demonstrated a clear separation between groups with different management strategies. The absolute control (T11) was positioned at the end of the Ci vector, in the lower right quadrant of the graph, evidencing an association with high internal CO_2_ values, which is frequently related to low photosynthetic activity driven by severe biochemical limitations (such as Rubisco inefficiency) due to nitrogen deficiency, as previously discussed. In contrast, the mineral nitrogen fertilization treatment (T12) and those with co-inoculation associated with re-inoculation (T9 and T10) showed greater proximity in the multivariate space, indicating similarity in the physiological profile of plants, with elevated CO_2_ assimilation A, higher stomatal conductance, photosynthetically active radiation, and shoot dry mass.

The variables with the greatest discriminant contribution were LNC, followed by A, PAR, and gs, which reinforces the relevance of nitrogen, whether supplied via mineral fertilization or symbiosis, as a key factor for the physiological performance of common bean. The absolute control, on the other hand, was negatively discriminated in the canonical axes, associating with low values of these variables, which reflects the physiological limitation imposed by the absence of nitrogen.

Therefore, CDA complements ANOVA results by confirming that, although some isolated variables did not show statistical differences among treatments, the set of physiological characteristics allows a clear distinction between different managements. This highlights the potential of co-inoculation with re-inoculation as an efficient strategy to promote integrated physiological improvements in bean plants.

#### 3.6.3 Agronomic variables

**Figure 8:**
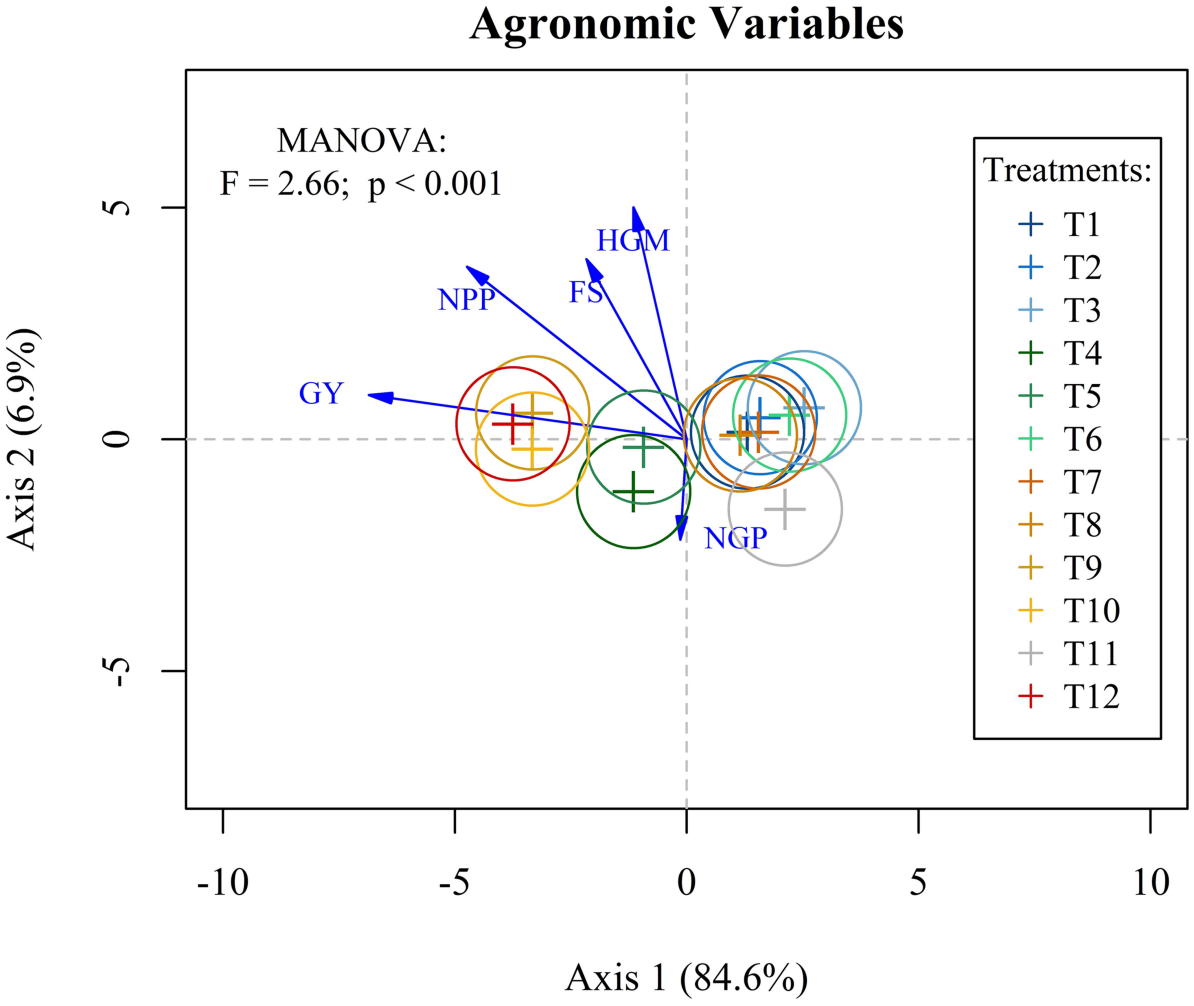
Canonical Discriminant Analysis (CDA) of common bean in response to inoculation techniques, co-inoculation, re-inoculation, and mineral fertilization based on agronomic traits: final stand (FS), number of pods per plant (NPP), number of grains per pod (NGP), 100-grain mass (HGM), and grain yield (GY). T1, Seed inoculation with *R. tropici*; T2, In-furrow inoculation with *R. tropici*; T3, Top-dressing inoculation with *R. tropici*; T4, Seed co-inoculation (*R. tropici* + *A. brasilense*); T5, In-furrow co-inoculation (*R. tropici* + *A. brasilense*); T6, Top-dressing co-inoculation (*R. tropici* + *A. brasilense*); T7, T1 + subsequent top-dressing re-inoculation; T8, T2 + subsequent top-dressing re-inoculation; T9, T4 + subsequent top-dressing re-inoculation; T10, T5 + subsequent top-dressing re-inoculation; T11, Absolute control (no N source); T12, Mineral N fertilization at sowing and top-dressing (100 kg N ha-1). The MANOVA results indicated a significant effect of treatments on morphophysiological variables (F = 3.98; p < 0.001), with confidence ellipses (95% level) representing group dispersion.

The CDA was employed as a complementary tool to the univariate analysis ANOVA, aiming to integrate the joint behavior of multiple agronomic variables in discriminating the evaluated treatments. The significance obtained by MANOVA confirmed that, when analyzed jointly, these variables allow for a robust distinction of experimental groups. The first two canonical axes explained 91.5% of the total variance together, with 84.6% attributed to Axis 1 and 6.9% to Axis 2 (**Figure 8**).

Vectors for GY, NPP, FS, and HGM were more strongly associated with Axis 1, evidencing their greater contribution to the multivariate separation among treatments. Conversely, NGP showed less influence, with projections close to the center, indicating less expressive variation among treatments for this variable. The projection of treatments in the canonical space revealed patterns consistent with ANOVA results, but with greater clarity regarding the relationship between treatments and the variable set. Treatments T9, T10, and T12 stood out by positioning themselves aligned with the GY vector, followed by contributions from NPP, FS, and HGM vectors, evidencing their superior performance in terms of integrated productivity and high agronomic efficiency. In contrast, treatments such as T3 and T11 occupied positions opposite to the productive vectors, confirming the inferior performance already verified in previous analyses.

Overall, CDA complements the univariate analysis by evidencing the discriminatory capacity of the variables as a set, highlighting the superiority of strategies combining co-inoculation with re-inoculation, especially in treatments T9 and T10. The clustering of these integrated biological treatments near the full mineral fertilization standard (T12) visually corroborates their efficacy in sustaining high yield components. This type of multivariate approach is essential to understand the complexity of agronomic effects, allowing for a more integrated assessment of plant response to symbiotic nitrogen supply and PGPB stimulation within sustainable agricultural systems.

## 4 Conclusion

Inoculation, co-inoculation, and re-inoculation techniques have demonstrated the potential to promote superior morphological, physiological, and agronomic performance in common bean crops.

Under the conditions of the present study, co-inoculation of *R. tropici* + *A. brasilense*, applied via seed or in-furrow, followed by top-dressing re-inoculation at the V4 stage, yielded the best results, achieving a mean grain yield of 3,042 kg ha^-1^. This was statistically equivalent to the yield obtained in the standard treatment with mineral nitrogen fertilization 3,124 kg ha^-1^ and approximately 67% superior to the mean yield of the control treatment.

Thus, co-inoculation associated with top-dressing re-inoculation demonstrates the potential to be a sustainable and technically viable alternative for increasing common bean productivity. Furthermore, future studies across different locations and growing seasons are recommended to foster more efficient and sustainable cropping systems.

## Data Availability

The raw data supporting the conclusions of this article will be made available by the authors, upon reasonable request to the corresponding author.
